# Impact of metallothionein-knockdown on cisplatin resistance in malignant pleural mesothelioma

**DOI:** 10.1038/s41598-020-75807-x

**Published:** 2020-10-29

**Authors:** Sabrina Borchert, Pia-Maria Suckrau, Robert F. H. Walter, Michael Wessolly, Elena Mairinger, Julia Steinborn, Balazs Hegedus, Thomas Hager, Thomas Herold, Wilfried E. E. Eberhardt, Jeremias Wohlschlaeger, Clemens Aigner, Agnes Bankfalvi, Kurt Werner Schmid, Fabian D. Mairinger

**Affiliations:** 1 Institute of Pathology, University Hospital Essen, University of Duisburg-Essen, Essen, Germany; 2 German Cancer Consortium (DKTK), Partner Site University Hospital Essen, Hufelandstrasse 55, 45122 Essen, Germany; 3 Department of Thoracic Surgery and Thoracic Endoscopy, Ruhrlandklinik, University Hospital Essen, University of Duisburg-Essen, Essen, Germany; 4 Department of Medical Oncology, West German Cancer Centre, University Hospital Essen, University of Duisburg-Essen, Essen, Germany; 5 Ruhrlandklinik, West German Lung Centre, University Hospital Essen, University of Duisburg-Essen, Essen, Germany; 6 Department of Pathology, Diakonissenkrankenhaus Flensburg, Flensburg, Germany

**Keywords:** Mesothelioma, Mesothelioma

## Abstract

Malignant pleural mesothelioma (MPM) is a rare, but aggressive tumor with dismal prognosis. Platinum-based chemotherapy is regularly used as part of multimodality therapy. The expression of metallothioneins (MT) has been identified as a reason for cisplatin resistance, which often leads to early therapy failure or relapse. Thus, knockdown of MT expression may improve response to cisplatin treatment. The MT gene- and protein expression of the MPM-cell lines MSTO-211H, NCI-H2052 and NCI-H2452 and the human fibroblast cell line MRC-5, as well as their sensitivity to cisplatin treatment have been evaluated. Knockdown of MT1A, 1B and 2A expression was induced by RNA interference. MT expression was measured using quantitative real-time PCR. An in vitro Assay based on enzyme activity was used to detect cell viability, necrosis and apoptosis before and after incubation with cisplatin. MT2A gene expression could be detected in all MPM cell lines, showing the highest expression in NCI-H2452 and NCI-H2052, whereas gene expression levels of MT1A and MT1B were low or absent. The immunohistochemically protein expression of MT-I/II reflect MT2A gene expression levels. Especially for MSTO-211H cell presenting low initial MT2A levels, a strong induction of MT2A expression could be observed during cisplatin treatment, indicating a cell line-specific and platin-dependent adaption mechanism. Additionally, a MT2A-dependent cellular evasion of apoptosis during cisplatin could be observed, leading to three different MT based phenotypes. MSTO-211H cells showed lower apoptosis rates at an increased expression level of MT2A after cisplatin treatment (from sixfold to fourfold). NCI-H2052 cells showed no changes in MT2A expression, while apoptosis rate is the highest (8–12-fold). NCI-H2452 cells showed neither changes in alteration rate of MT2A expression nor changes in apoptosis rates, indicating an MT2A-independent resistance mechanism. Knockdown of MT2A expression levels resulted in significantly induced apoptotic rates during cisplatin treatment with strongest induction of apoptosis in each of the MPM cell lines, but in different markedness. A therapeutic meaningful effect of MT2A knockdown and subsequent cisplatin treatment could be observed in MSTO-211H cells. The present study showed MT2A to be part of the underlying mechanism of cisplatin resistance in MPM. Especially in MSTO-211H cells we could demonstrate major effects by knockdown of MT2A expression, verifying our hypothesis of an MT driven resistance mechanism. We could prove the inhibition of MT2A as a powerful tool to boost response rates to cisplatin-based therapy in vitro. These data carry the potential to enhance the clinical outcome and management of MPM in the future.

## Introduction

Malignant pleural mesothelioma (MPM) is a highly aggressive and mainly asbestos-related tumor, arising from pleural cavities^1,2^. The state-of-the-art systemic treatment of unresectable and advanced MPM is chemotherapy including a combination of cis- or carboplatin and the antifolate pemetrexed^3,4^. However, MPM patients have poor prognosis with a median survival of approximately 14 months^5^. The response rate of MPM to single-agent cisplatin-based antiproliferative treatment is merely 14%, in the combination with pemetrexed response rates are up to 45%^6^.

Several studies have been involved in searching for biomarkers in DNA repair pathways, trying to elucidate the causes of the resistance mechanism, attempting to improve clinical management^1,7–10^. However, many alterations existing in individual patients constitute a serious obstacle for the identification of biomarkers for patient stratification^11,12^.

Metallothioneins (MT) are cysteine-rich, low-molecular-mass proteins involved in numerous processes in cells including proliferation and apoptosis^13,14^. The most commonly expressed metallothioneins MT1 and MT2 are two of the four human isoforms^15,16^. Beside functions of regulating zinc homeostasis^17–19^, MTs can have heavy metal detoxifying effects, as they bind heavy metals via thiol groups on cysteine residues and oxidize them^16,20^. Against this background it might be suggested that MTs may cause drug resistance by binding cisplatin, oxidize and thus, inactivate it. In a previous study, we displayed a negative correlation between progression-free survival and expression of MT1/2 in 105 MPM patients^21^, maybe indicating cisplatin resistance is induced by MT-overexpression in tumor cells^21^. We also could suggest that miRNA expression of miR-566 is associated with MT1A and MT2A immunoexpression in clinical samples^21^.

In this study, we investigated the influence of MT1A, MT1B and MT2A on cisplatin-sensitivity in human MPM cell lines. We induced knockdown of MT expression via short interfering RNA (siRNA) and analyzed cells for apoptosis, necrosis and viability during cisplatin treatment. Additionally, we generated FFPE specimens of the treated cell lines for gene- and protein expression analysis. This study might deepen the understanding of the underlying biology of protein expression patterns to stratify chemotherapy responder from non-responders.

## Results

### Expression of metallothioneins is cell type-specifically induced by cisplatin

**Figure 1:**
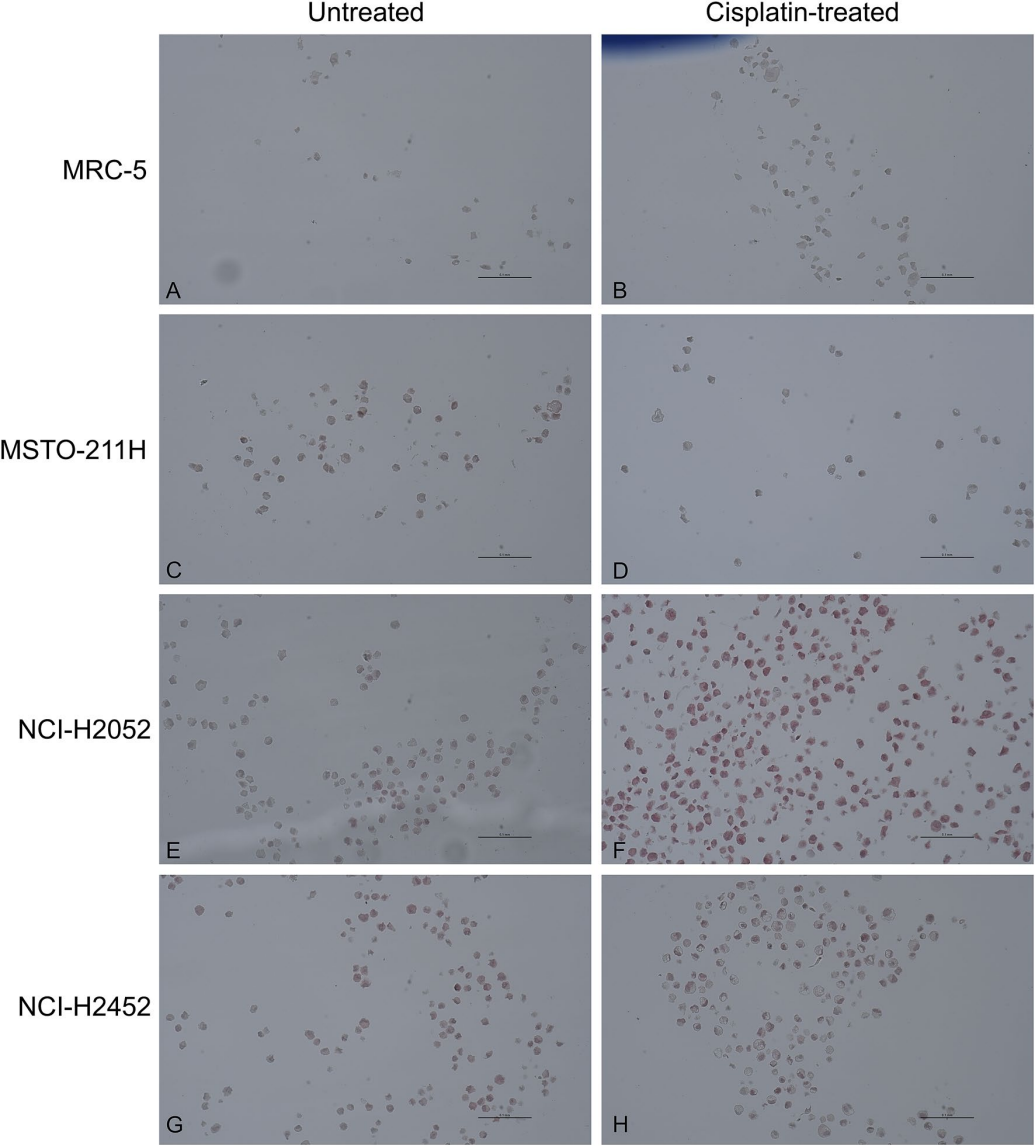
Immunohistochemically staining of MT1/2 before and after cisplatin treatment of cell lines. MPM cell lines MSTO-211H, NCI-H2052 and NCI-H2452, as well as the lung fibroblast cell line MRC-5 were stained for MT1/2. On the basis of these figures, scoring of MT expression was performed as shown in Table 1. Cisplatin-treated cells were exposed to 10 µM cisplatin for 24 h and then harvested for formalin fixation and FFPE embedding. MRC-5 cells sowed no MT expression, while MSTO-211H showed minimal expression and both cell lines did not show changes in MT expression after cisplatin treatment. Both cell lines NCI-H2052 and NCI-H2452 showed moderate MT-expression when untreated and an increased expression after cisplatin treatment.

Immunohistochemical staining with MT1/2 was performed for all four cell lines before and after treatment with cisplatin (Fig. 1). Based on immunohistochemical staining of cells, MT could not be detected in MRC-5 cells (Table 1). MSTO-211H cells showed a small number of MT-positive cells (Score 1) and did not change after cisplatin treatment. Both the NCI-H2052 and NCI-H2452 cell lines showed a score of 2 before and a score of 3 (> 50% of MT-positive cells) after treatment with cisplatin. Possible localizations of MT-staining (nucleus, cytoplasm or both) are depicted in Suppl. Figure 1.

**Table 1:** Scores of metallothionein expression specified by immunohistochemical staining.

Cell line	Score (before cisplatin treatment)	Score (after cisplatin treatment)
MRC-5	0	0
MSTO-211H	1	1
NCI-H2052	2	3
NCI-H2452	1	2

By comparing immunohistochemically with qPCR results, no correlation regarding MT1A expression could be found. MT1B showed proportional correlations, indicating that a strong mRNA expression of MT1B resulted in higher protein yields and thus correlated with a higher Score (p = 0.0012, Suppl. Figure 2). This also applies to MT2A, as a higher ∆Cp-value correlated with higher scores (p < 0.0001).

**Figure 2:**
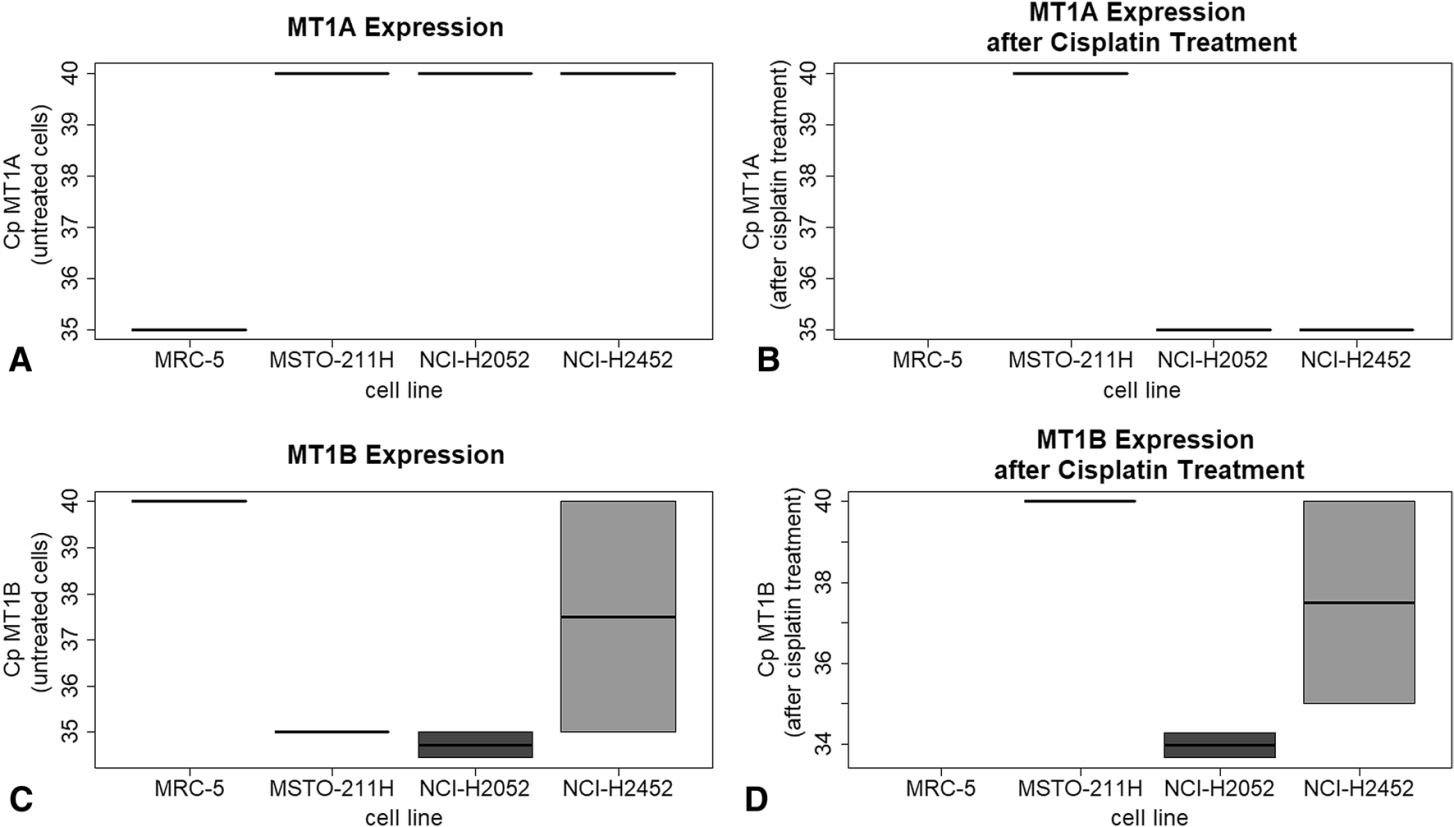
Expression of MT 1A and MT1B in all four cell lines before and after treatment with cisplatin. The qPCR analyses showed that MT1A (**A**,**B**) is not expressed in untreated cells but low expressed in NCI H2452 and NCI-H2052 cells after cisplatin treatment. MT1B (**C**,**D**) was also low expressed and changed only in MSTO-211H cells after cisplatin treatment.

**Figure 3:**
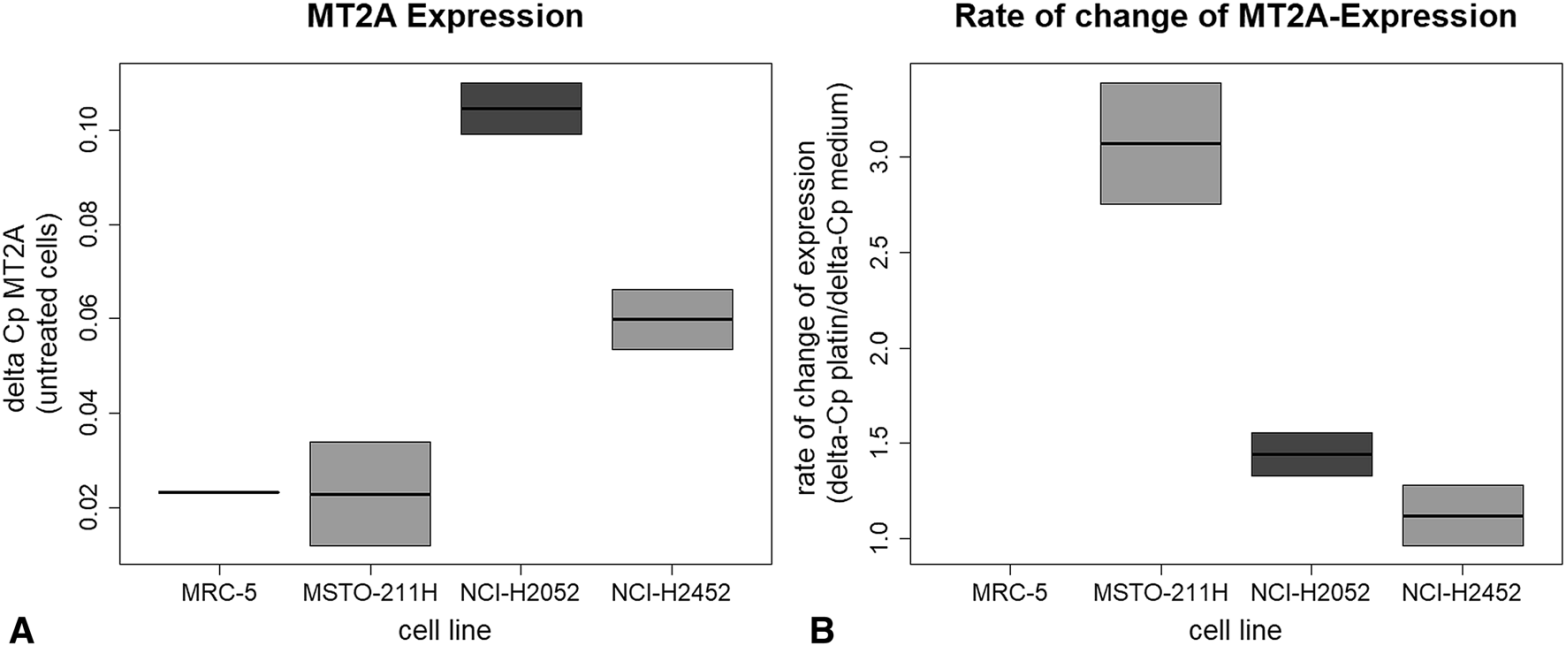
MT2A Expression and rate of change of MT2A-expression before and after Cisplatin treatment. MSTO-cells showed a threefold increase of MT2A expression after treatment with cisplatin (**B**). In H2052 cells, MT2A shows a basically higher expression than other cell lines (**A**) but does not change after cisplatin treatment.

Metallothionein 1A and 1B showed heterogeneous gene expression levels in investigated cell lines (Fig. 2). Except for the non-tumorous control cell line MRC-5, no expression of MT1A could be detected. After treatment with cisplatin, cell lines NCI-H2452 and NCI-H2052 showed minimal expression. In contrast, MT1B is basally expressed in cell lines, except for the control cell line MRC-5, without cisplatin treatment. After cisplatin treatment, no expression of MT1B could be detected in MSTO-211H, while NCI-H2052 cells showed increased expression levels. NCI-H2452 cells showed no altered MT1B gene expression. MT2A is expressed in all MPM cell lines (Fig. 3A). Interestingly, cisplatin untreated MSTO-211H cells showed lowest gene expression level of MT2A as compared to NCI-H2052 and NCI-H2452 cells. However, after cisplatin treatment, this cell line showed the highest alteration rate of MT2A expression (Fig. 3B).

**Figure 4:**
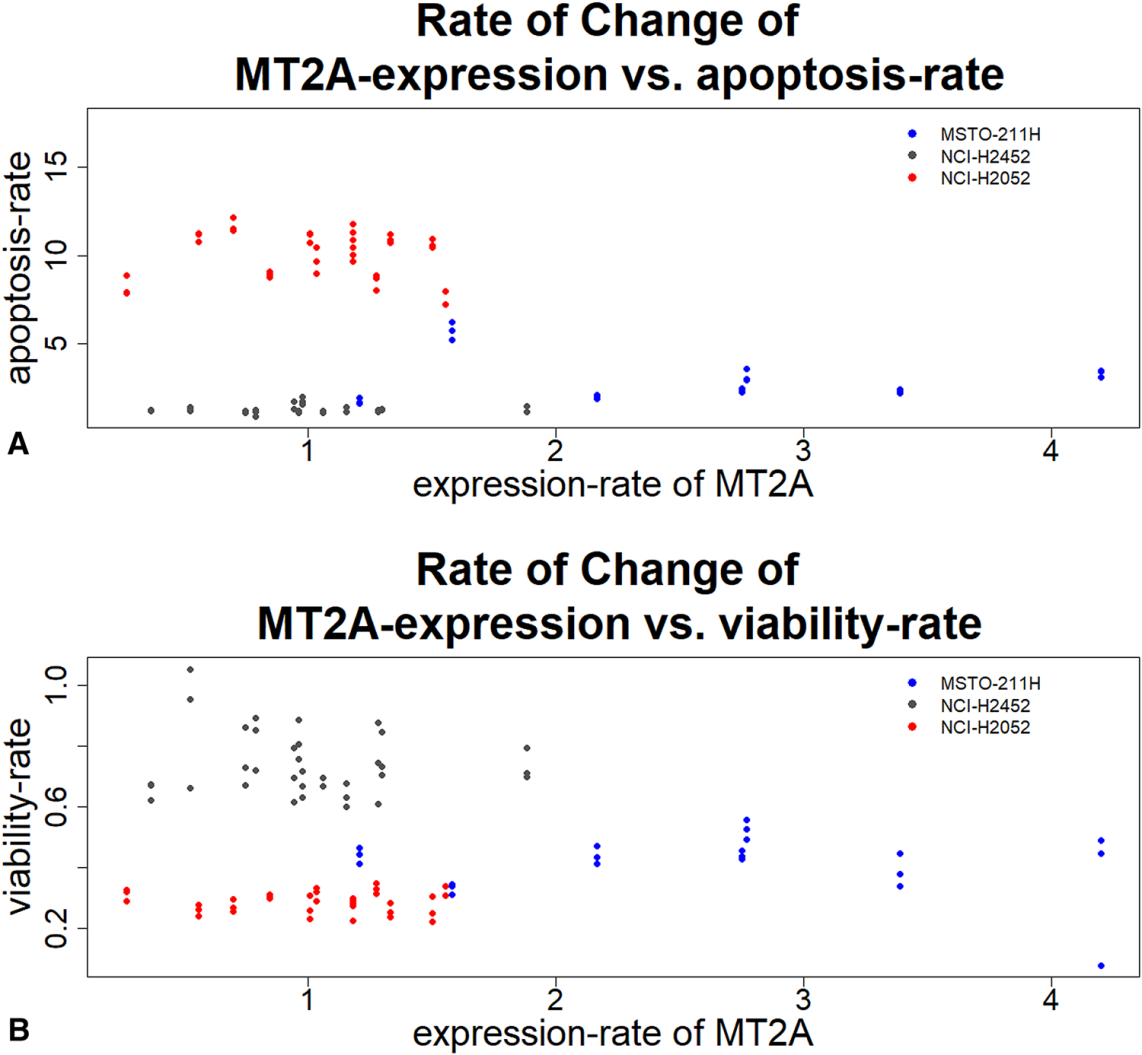
The rate of change of MT2A-expression vs. Apoptosis rate (**A**) and vs. viability-rate (**B**). Three phenotypes could be observed. MSTO-211H cells showed increased gene expression of MT2A and decreased apoptosis rates after cisplatin treatment. NCI-H2452 cells showed neither response to cisplatin treatment nor changes in apoptosis rates. NCI-H2052 cells showed the highest apoptosis rates, while MT2A expression was not changed after cisplatin treatment.

Furthermore, MSTO-211H cells showed lower apoptosis rates at an increased expression level of MT2A after cisplatin treatment (from sixfold to fourfold) by comparing the alteration rate of MT2A expression with the apoptosis rate of MPM cell lines (Fig. 4). NCI-H2052 cells showed no changes in MT2A expression, while apoptosis rate is the highest (8–12-fold), compared to the other cell lines. NCI-H2452 cells showed neither changes in alteration rate of MT2A expression nor changes in apoptosis rates and, in addition, showed the lowest apoptosis rates after cisplatin treatment (1.5-fold).

### MT2A influences apoptosis rates specifically

By plotting apoptosis rates and the rate of change of MT2A expression against each other, three phenotypes could be observed (Fig. 4). NCI-H2052 and NCI-H2452 cells showed no changes in MT2A expression during cisplatin treatment. However, NCI-H2052 cells showed higher apoptosis rates than NCI-H2452 cells. MSTO-211H cells increases MT2A expression 3 to 4-fold after cisplatin treatment. Higher MT2A expression levels result in lower apoptosis rates measured in this cell line. Therefore, the three cell lines showed different phenotypes, regarding MT-expression and the sensitivity against cisplatin.

### MT2A knockdown shows phenotype-specific influence on apoptosis induction during cisplatin treatment

**Figure 5:**
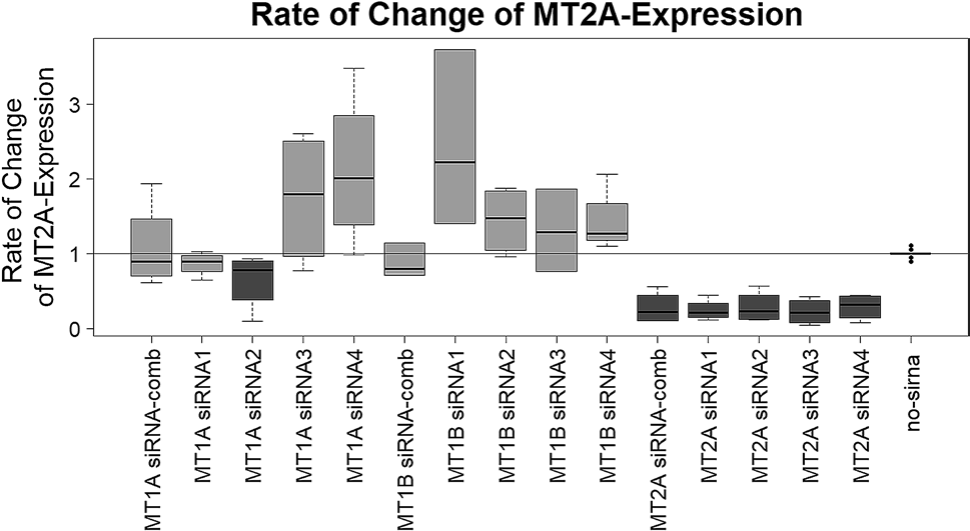
Rate of change of MT2A expression of all three MPM cell lines tested. As expected, most of siRNAs targeting MT1A and MT1B did not reduce MT2A expression. However, siRNA no. 2 targeting MT1A showed decreased MT2A expression to 80%. Expression of MT2A was reduced to 40–50%. Shapiro-Wilks-test revealed a non-parametric distribution. Therefore, Wilcoxon Mann–Whitney rank sum test was applied. P-values see Table 2. P-values are shown in Table 2.

The phenotype-specific effect of apoptosis induction during cisplatin treatment arising from MT2A-knockdown was analyzed by assessing gene expression of MT2A after knockdown via qPCR. In fact, we could reduce the expression of MT2A significantly as shown in Fig. 5 (p-values in Table 2). However, siRNA2 targeting MT1A, also reduced expression of MT2A, whereas other siRNAs targeting MT1A or MT1B primarily lead to increased expression of MT2A.

**Table 2:** P-values of the rate of change of MT2A-expression.

Treatment	p-value	Adj. p.value
siRNA-comb MT2A	5.289 × 10^–7^	9.917 × 10^–7^
siRNA1 MT2A	5.289 × 10^–7^	9.917 × 10^–7^
siRNA2 MT1A	5.289 × 10^–7^	9.917 × 10^–7^
siRNA2 MT2A	5.289 × 10^–7^	9.917 × 10^–7^
siRNA3 MT2A	5.289 × 10^–7^	9.917 × 10^–7^
siRNA4 MT1A	1.997 × 10^–7^	9.917 × 10^–7^
siRNA4 MT1B	5.289 × 10^–7^	9.917 × 10^–7^
siRNA4 MT2A	5.289 × 10^–7^	9.917 × 10^–7^
siRNA2 MT1B	1.582 × 10^–5^	2.637 × 10^–5^
siRNA1 MT1B	1.784 × 10^–5^	2.677 × 10^–5^
siRNA1 MT1A	2.560 × 10^–5^	3.491 × 10^–5^
siRNA-comb MT1B	6.050 × 10^–4^	7.562 × 10^–4^
siRNA3 MT1A	5.806 × 10^–3^	6.699 × 10^–3^
siRNA3 MT1B	1.305 × 10^–2^	1.398 × 10^–2^
siRNA-comb MT1A	9.857 × 10^–1^	9.857 × 10^–1^

Significant reduction of MT2A expression could be detected with a median estimate of 73% (confidence interval (95%) between 64 and 79%). P-values were FDR-adjusted.

**Figure 6:**
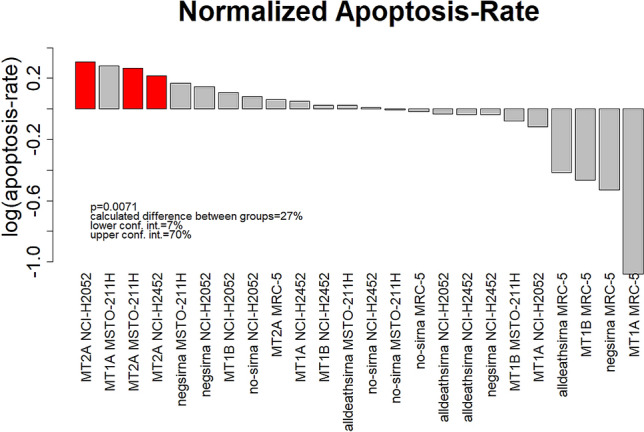
Normalized apoptosis rates were plotted against the implemented treatment. Highest apoptosis rates, after cisplatin treatment, were detected in MPM cells, when knockdown of MT2A expression was induced (red bars). Wilcoxon Mann–Whitney rank sum test was applied.

The waterfall plot, showing log2-values of normalized apoptosis rates, illustrates the strong influence of MT2A knockdown in all three MPM cell lines (Fig. 6). Knockdown of this target lead to the highest apoptosis rates in cell lines.

## Discussion

In a previous study, we found expression of metallothionein being predictive regarding response to cisplatin therapy in 105 MPM patients^21^. In the current study, we investigated the expression of metallothionein MT1A, MT1B and MT2A in selected MPM cell lines and the influence of cisplatin and siRNAs on MT-expression. Furthermore, we aimed to find associations between MT-expression and sensitivity to cisplatin-based chemotherapy in investigated cell lines.

Based on our hypothesis of a decisive role of metallothioneins in cellular response to platinum-based therapeutic approaches, the main aim of the present study was to proof a MT-based effect on induction of apoptosis via cisplatin. Therefore, we artificially reduced MT gene expression using specific siRNAs. We could show elevated response to cisplatin after knockdown of MT2A expression in all four MPM cell lines (Fig. 6). MT knockdown alone did not influence cell state, proving a plain therapy associated effect. However, only MSTO-211H cells showed significant changes between MT2A expression levels before and after cisplatin treatment. Therefore, knockdown of MT2A expression and subsequent cisplatin treatment lead to highest effects in this cell line. These findings underline the key role of MT2A in cellular resistance against platinum compounds. The high expression of MT2A in MSTO-211H cells after cisplatin treatment and subsequent reduction of apoptosis rates further support our hypothesis. This may become of great clinical interest, as functional inhibition of MTs is a promising approach to increase patients’ response rates and thereby improve patients’ clinical management.

The human fibroblast cell line MRC-5 was used as healthy control instead of more commonly used immortalized mesothelial cell lines like MET-5A or LP-9. They derive from the same cotyledon (mesoderm), like mesothelial cells and show a more comparable gene expression pattern to naïve pleura compared to the above-mentioned mesothelial cell lines. Moreover, it was preferred, as the e.g. control cell line MET-5A was SV40-immortalized and therefore shows altered culture performance^22^. The TERT1-immortalized cell line LP-9 would be another option for using as control cell line^23^. However, the use of this cell line as control has to be investigated and validated in further studies.

In the present study, we could proof earlier finding in clinical samples, indicating MTs crucial role in cellular response to cisplatin. Previous studies showed negative correlations of progression-free and overall survival and MT-expression^21^. In line with these findings, different cell lines showed different sensitivity against treatment with platinum compounds. In summary, three different phenotypes regarding MT2A gene expression and its change rates as cellular response to platinum treatment could be observed:

1. NCI-H2052 cells, showing very low changes in MT2A expression but high apoptotic rates
2. The MSTO-211H cells with increased MT2A expression levels during cisplatin treatment, but high apoptotic rates after knockdown of MT2A expression
3. The NCI-H2452 cells, showing neither changes in MT2A expression nor changes in apoptotic rates.

Depending on the MT phenotype, knockdown of MT expression has different influence on beneficial effects of different response rates to cisplatin in cell lines. For MSTO-211H-cells, which showed a strong induction of MT2A expression, building up a protective barrier against cisplatin, the observed effect may be of profound clinical significance. Similar observations have been made in different tumor entities including renal carcinoma and urothelial carcinoma^24,25^.

Most of siRNAs targeting MT1A and MT1B lead to increased MT2A expression. As a possible explanation for this observation, we hypothesized a positive feedback loop induced by artificially lowered MT-I levels. De Francisco et al. also assume a coordinated transcriptional regulation between MT-isoforms^26^. It could be hypothesized, that knockdown of MT1A or MT1B did not induce apoptosis caused by upregulation of MT2A, being the main MT inducing phenotype-specific overexpression during cisplatin treatment^27,28^. In line with this, MT2A is the main isoform being responsible for heavy metal detoxification whereas the others are mainly involved in a variety of different cellular processes, for example regulation of cellular zinc homeostasis^19,29,30^.

The poor response on cisplatin by NCI-H2452 cells indicates different resistance mechanisms playing a role in this cell line. One resistance mechanism could be the BAP1 mutation in NCI-H2452 cells, as we could show apoptotic effects in this cell line by treatment with PARP1-inhibitor olaparib^31^.

Surprisingly, knockdown of MT1A using siRNA2 resulted in comparably high normalized elevations in apoptosis rates as MT2A knockdown (Fig. 6). This is contractionary to our observation, that knockdown of MT1A reduced sensitivity of the cells to platin-based treatment, as those reductions of MT1A expression result in elevated MT2A-levels. By analyzing these data more profoundly, we could prove off-target effects leading to significant reduced MT2A levels (Fig. 5). Therefore, the elevated apoptosis rates could be traced back to the MT2A knockdown driven effect. Interestingly, this unspecific off-target effect of this siRNA only causes significantly clinical effects in MSTO-211H cells (Suppl. Figure 3).

The expression of MTs was analyzed on RNA- and protein levels. Several studies revealed that MT-expression increases after treatment with heavy metals such as cisplatin^32^. We could observe cell-type specific MT expression as well as a correlation between the amount of protein and mRNA. The protein expression scores specified by immunohistochemistry showed differing changes after cisplatin treatment compared to the alteration rates of gene expression gained from qPCR results. The reason for this could be the combined evaluation of both MT1 and MT2 protein both targeted simultaneously by the antibody used in this study. Additionally, qPCR is a quantitative method and therefore is more precise than visual examination by showing a higher dynamic range. Furthermore, mRNA quantity may not to be proportional to protein amounts.

MT1A showed no detectable gene expression level in all untreated MPM cell lines. However, as response to cisplatin treatment, gene expression levels of MT1A increased significantly.

It still shows very low gene expression levels, but the triplicate measurement showed clear and robust signals with minimal standard deviations around Cp = 35. Nobeyama et al. showed that MT1A expression could be regulated via methylation of 5′ MT1A CpG islands in melanoma^33^. It might be assumed, that demethylation of the MT1A locus may indicate a first cellular response to cisplatin, causing this observation. Methylation changes induced by cisplatin have also been observed in a study of Flanagan et al.^34^ and thus it could be assumed that MT1A may also be affected by methylation. Interestingly, this effect could not be observed for MT1B. For MSTO-211H, more likely a loss of expression was detected.

The results of MT2A gene expression, as well as knockdown experiments showed that MT2A seems to play a crucial role in these cell lines. Nevertheless, MT1B and MT2A also showed significant correlations between gene expression and protein expression scores.

In fact, MT-expression plays a crucial role regarding cisplatin-resistance. Nevertheless, MT-expression could not be used as a single biomarker but in combination with other factors, could provide important details for patients’ prognosis and clinical management. Regarding further biomarkers, microRNA-31 could be of great interest, as it is assumed to promote chemo sensitivity and may be a prognostic marker for chemotherapy^35^. Additionally, the human copper transporter 1 (hCTR1) also may influence cisplatin-resistance, as it also could bind cisplatin. Knockdown of the expression of this transporter may lead to higher cisplatin-resistance. In this case, therapeutic increase of hCTR1-expression may lead to higher sensitivity to cisplatin-therapy.

## Conclusion

In this in vitro study, knockdown of MT2A-expression revealed three cellular phenotypes regarding response to cisplatin. Especially in MSTO-211H cells we could demonstrate significant effects by knockdown of MT2A expression, supporting our hypothesis of an MT driven resistance mechanism. Inhibition of MT2A may be a promising approach to improve response to cisplatin-based therapy regimens. Conceivable approaches concerning therapeutic inhibition of MT2A in MPM patients could be the encapsulation of siRNA into biological membranes or nanocells^36^. Concerning that matter, Van Zandwijk et al. showed promising results during a phase I study in 2017 by using miRNA containing minicells known as EnGeneIC Dream Vectors (EDVs)^37,38^.

In conclusion, this might lead to an improved clinical management associated with increased survival and better clinical outcome in MPM patients in the future.

## Material and methods

### Study design

**Figure 7:**
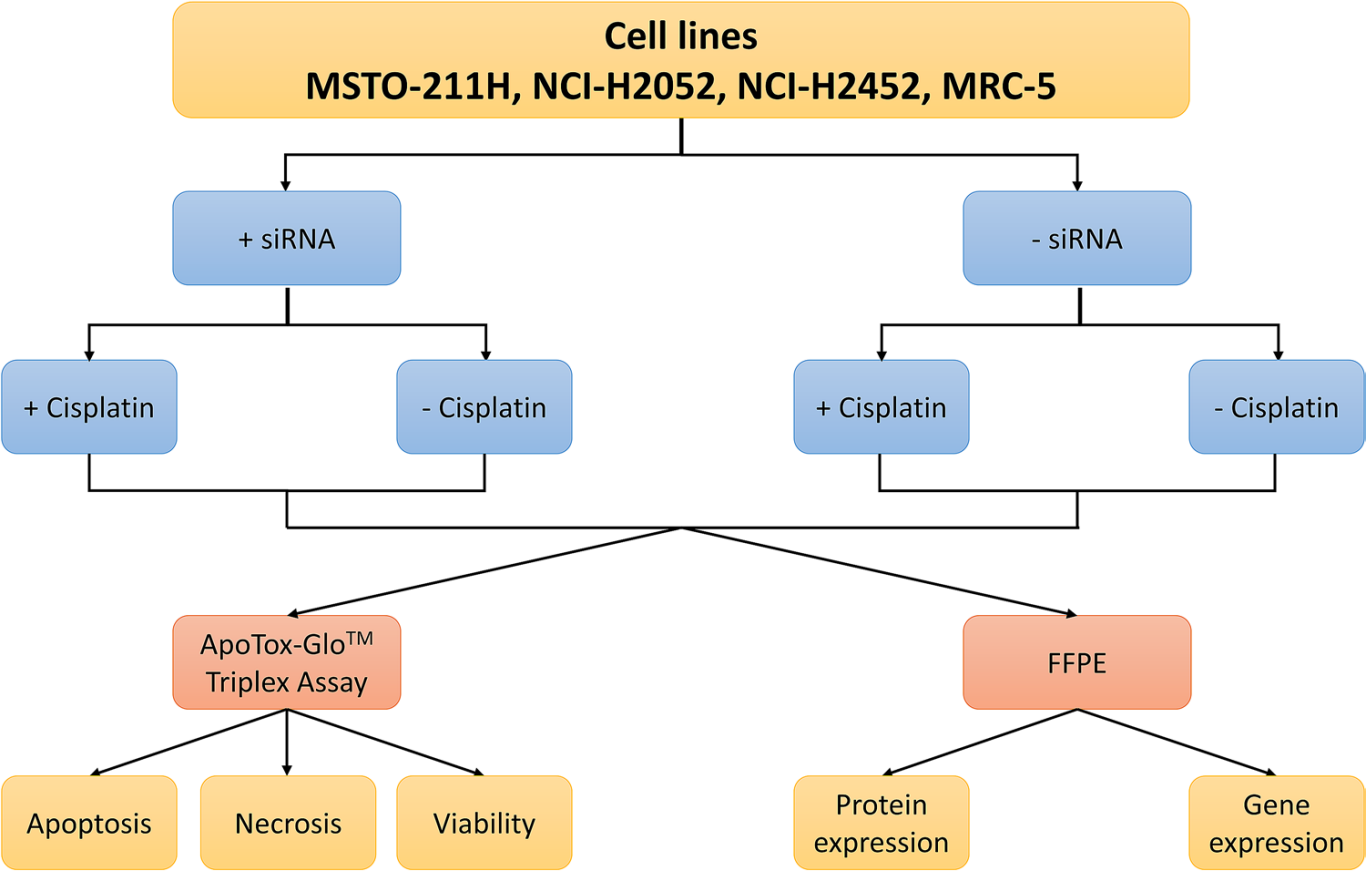
Study design of siRNA knockdown-experiments. Cell lines were treated with siRNA for knockdown of MT1A, MT1B and MT2A expression. Cells were treated with cisplatin and, after 48 h of incubation, were analyzed for apoptosis, necrosis and viability. Additionally, FFPE specimens were generated for gene- and protein expression analysis. As we generated one FFPE specimen for each condition in each cell line.

As described in Fig. 7, we investigated the influence of metallothionein on cisplatin resistance in MPM cells via knockdown of MT gene expression and subsequent detection of apoptosis, necrosis or viability of cells. In addition, we generated formalin-fixed, paraffin-embedded (FFPE) specimens of the treated or untreated cell lines to evaluate protein expression via immunohistochemistry (IHC) and gene expression via quantitative PCR (qPCR). Knockdown of MT1A, MT1B and MT2A was performed by using siRNA. For each target, four siRNAs were available (see chapter “Treatment of MPM cell lines with cisplatin and siRNAs”). In our cell lines, we tested each single siRNA and combined siRNAs for one target. For this study, MSTO-211H, NCI-H2052 and NCI-H2452 cells, as well as the healthy human lung-fibroblast cell line MRC-5 were evaluated. As we generated one FFPE-specimen for each cell line (4 cell lines) for each condition (siRNA: 4 siRNAs + combination for targets MT1A, MT1B and MT2A; cisplatin-treated or untreated) in biological triplicate, 12 (conditions) * 4 (cell lines) * 3 (targets) * 3 (triplicate) = 432 FFPE specimens were generated.

### Cell culture

MPM cell lines MSTO-211H (pemetrexed-sensitive, biphasic^39^) and NCI-H2052 (cisplatin-sensitive, sarcomatoid^39^) as well as the cell line NCI-H2452 (*BAP1*-mutant, cisplatin-resistant^40^, epithelioid^41^) were cultured in Roswell Park Memorial Institute (RPMI)-1640 medium (Thermo Fisher Scientific, Massachusetts), USA. The human lung-fibroblast cell line MRC-5 was used as control cell line. MRC-5 cells were cultured in Minimal Essential Medium (Thermo Fisher Scientific). All culture media were supplemented with 10% fetal calf serum and 1% penicillin and streptomycin (Thermo Fisher Scientific).

### Treatment of MPM cell lines with cisplatin and siRNAs

The cells were analyzed for apoptosis, viability and necrosis during treatment with cisplatin (Selleckchem, Houston, USA). In addition, the effect of siRNAs (FlexiTube siRNA, Qiagen, Hilden, Germany) targeting MT1A, MT1B and MT2A was tested for each cell line.

For the treatment of cells, 10,000 cells per well in a 96-well plate were applied. To evaluate the effect of downregulation of MT1A, MT1B or MT2A, 5 nM FlexiTube siRNA was added to cells referring to the reversed transfection protocol (Qiagen). 0.75 µl per well of HiPerFect Transfection Reagent (Qiagen) was applied for reversed transfection. AllStars HS Cell Death Control siRNA (Qiagen) was used for positive control and AllStars Negative Control siRNA (Qiagen) was used for negative control.

After 12 h of incubation, 10 µM of cisplatin were added to cells. The concentration was estimated for cisplatin by reviewing the literature^42^. As DMSO was used to solubilize cisplatin, 0.08% DMSO, the same concentration of DMSO in cisplatin-treated cells, was given to cells for control purposes. After 48 h of incubation with cisplatin, cells were analyzed by using the ApoTox-Glo^®^ Triplex Assay.

### ApoTox-Glo™ Triplex-Assay

Viability, necrosis and apoptosis of cells were analyzed by the ApoTox-Glo^®^ Triplex Assay kit (Promega, Wisconsin, USA). 50 µl of Digitonin (30 µg/ml, Selleckchem), added to the cells in a separate well 15 min before measurement, served as positive control for decreased viability to measure a decrease of cellular viability of 100%. 50 µl of Digitonin (30 µg/ml) was added to cells 2 h before measurement, serving as positive control for cytotoxicity. 50 µl of Staurosporine (10 µM, Selleckchem) served as positive control for apoptosis and was given to the cells 3 h before measurement. Reactions were measured by using a luminometer (Glo Max Multi + Detection System; Promega).

Changes in cell state were calculated as percentage of signal gained by the positive control (100% apoptosis, cytotoxicity or decrease in viability) normalized to the baseline (untreated cells).

### Cell harvesting and FFPE embedding

Cells were cultured in T-175 flasks (4 × 10^6^ cells/flask) and treated in the same manner as with the cells being analyzed using the ApoTox-Glo^®^ Triplex assay. After 48 h of incubation from cisplatin treatment, cells were harvested by incubating with 0.25% Trypsin–EDTA (Thermo Fisher Scientific) for 2–5 min, centrifuged for 3 min at 300×*g* and then fixed in 8 ml of 4.5% Formalin (Otto Fischar GmbH & Co. KG, Saarbrucken, Germany).

Fixed cells were centrifuged at 800×*g* for 10 min. The cell pellet was transferred into a 1.5 ml reaction tube and stained with one drop of Eosin-G solution (0.5%, Carl Roth, Karlsruhe, Germany). The tubes were centrifuged at 4000×*g* for 2 min. The supernatant was removed by a pipette. The pellet was mixed with ca. 1 ml of 1% agarose (Merck KGaA, Darmstadt, Germany). After solidification (ca. 10 min), the gel was bisected and transferred into embedding cassettes. Cassettes were stored in 4.5% Formalin until dehydration. After dehydration, cassettes were embedded in paraffin and stored at room temperature.

### RNA-isolation and quantification

RNA-purification from 3 to 8 10 µm thick FFPE sections was performed by using the Maxwell RSC RNA FFPE Kit (Promega), according to the manufacturer’s instructions. Prior to isolation, sections were stored at − 20 °C in 1,5 ml tubes until use for RNA isolation as this procedure resulted in higher RNA yields as shown before^43^. RNA was eluted in 50 µl RNase-free water and stored at − 80 °C.

The concentration of RNA was determined via fluorometric quantification (Qubit, Thermo Fisher Scientific) using the RNA Broad range assay kit according to the manufacturer’s instructions. 1 µl of each isolated RNA sample was applied for measurement.

### RNA-precipitation and reverse transcription

Precipitation of RNA was performed to gain the required RNA concentration for reverse transcription. Therefore, 5 µl of sodium acetate (3 M) and 125 µl ethanol (100%, on dry ice) was added to each probe. After 1.5 h of incubation on dry ice, probes were centrifuged at full speed and 4 °C for 45 min. Supernatant was removed, probes were dried and subsequently solubilized with RNase-free water.

RNA concentration was determined again via fluorometric quantification (Qubit). Reverse transcription was performed by using the RevertAid First Strand cDNA Synthesis Kit (Thermo Fisher Scientific) according to the manufacturer’s instructions. 11 µl of concentrated RNA (1 µg total) was applied. Random-Hexamer-Primer was added to the master-mix. After cDNA-synthesis, probes were stored at − 80 °C.

### Quantitative real-time PCR

For expression analysis of MT1A, MT1B and MT2A, quantitative real-time PCR (qPCR) with 40 cycles was performed according to the manufacturer’s instructions (TaqMan^®^ Universal PCR Master Mix User Guide, 2014, Thermo Fisher Scientific). 1 µl of cDNA (50 ng per reaction) was applied to each reaction. PCR reaction was analyzed by using the Light Cycler 480 Instrument II (Roche, Mannheim, Germany). For each probe, expression of MT1A, MT1B and MT2A were measured in triplicate. Actin-beta (ACTB) and glycerinaldehyde-3-phosphate-dehydrogenase (GAPDH) served as reference genes. The quantification of mRNA was calculated by using the 2^−ΔΔ^Cp method.

All PCR samples were analyzed by following the MIQE-guidelines^44^.

### Immunohistochemistry

Immunohistochemistry of cisplatin-treated and untreated, FFPE-embedded cells was performed according to standard protocols using an automated stainer (Ventana Discovery XT, Munich, Germany) and stained for MT1 and MT2 (monoclonal primary antibody MT1/MT2 clone 9, Agilent, Santa Clara, CA, USA). A semi quantitative scoring system, based on percentage of positive stained cells was used (score 0: no immunohistochemically signal, score 1: 1–5% MT-positive cells, score 2: 6–50% MT-positive cells, score 3: > 50% MT-positive cells)^21^.

### Statistical analysis

The R statistical programming environment (v3.2.3) was used for statistical and graphical analyses.

Before starting the analysis, the Shapiro–Wilks-test was applied to test for normal distribution of the data. Based on these results, either a parametric or non-parametric test was performed. For dichotomous variables, either the Wilcoxon Mann–Whitney rank sum test (non-parametric) or two-sided students t-test (parametric) was applied. In case of ordinal variables with more than two groups, either the Kruskal–Wallis test (non-parametric) or ANOVA (parametric) was used to detect group differences.

Double dichotomous contingency tables were analyzed by using Fisher’s Exact test. To test the dependency of ranked parameters with more than two groups, the Pearson’s Chi-squared test was performed. Correlations between metric variables were tested by using the Spearman’s rank correlation test as well as the Pearson's product moment correlation coefficient for linear modelling.

Due to the multiple statistical testing, all p-values were adjusted by using the false discovery rate (FDR). The level of statistical significance was defined as p ≤ 0.05 after adjustment.

### Ethical approval

This study is based only on commercially available cell lines and thus there is no additional statement in relation to ethical vote of ethical committee of the University Hospital Essen.

## Supplementary information

Supplementary Information.

## Data Availability

The datasets used and/or analyzed during the current study are available from the corresponding author on reasonable request.
