# Supplementary material for: Impact of metallothionein-knockdown on cisplatin resistance in malignant pleural mesothelioma
Source: Sci Rep. 2020 Oct 29;10:18677. doi: 10.1038/s41598-020-75807-x (PMC7596082; doi:10.1038/s41598-020-75807-x)
Supplement: Supplementary file 1 — Supplementary Information. [file 41598_2020_75807_MOESM1_ESM.pdf]

Impact of Metallothionein-Knockdown on Cisplatin Resistance in Malignant Pleural Mesothelioma

Sabrina Borchert[1,2*,#], Pia-Maria Suckrau[1#], Robert F. H. Walter[1,2], Michael Wessolly[1,2], Elena Mairinger[1,2], Julia Steinborn[1,2], Balazs Hegedus[3], Thomas Hager[1,2], Thomas Herold[1,2], Wilfried E.E. Eberhardt[4,5], Jeremias Wohlschlaeger[1,6], Clemens Aigner[3], Agnes Bankfalvi[1,2], Kurt Werner Schmid[1,2], Fabian D. Mairinger[1,2]

[1]Institute of Pathology, University Hospital Essen, University of Duisburg-Essen, Essen, Germany

[2]German Cancer Consortium (DKTK), Partner Site University Hospital Essen, Hufelandstrasse 55, 45122 Essen, Germany

[3]Department of Thoracic Surgery and Thoracic Endoscopy, Ruhrlandklinik, University Hospital Essen, University of Duisburg-Essen, Essen, Germany

[4]Department of Medical Oncology, West German Cancer Centre, University Hospital Essen, University of Duisburg-Essen, Essen, Germany

[5]Ruhrlandklinik, West German Lung Centre, University Hospital Essen, University of Duisburg-Essen, Essen, Germany

[6]Department of Pathology, Diakonissenkrankenhaus Flensburg, Flensburg, Germany

[#] Authors contributed equally

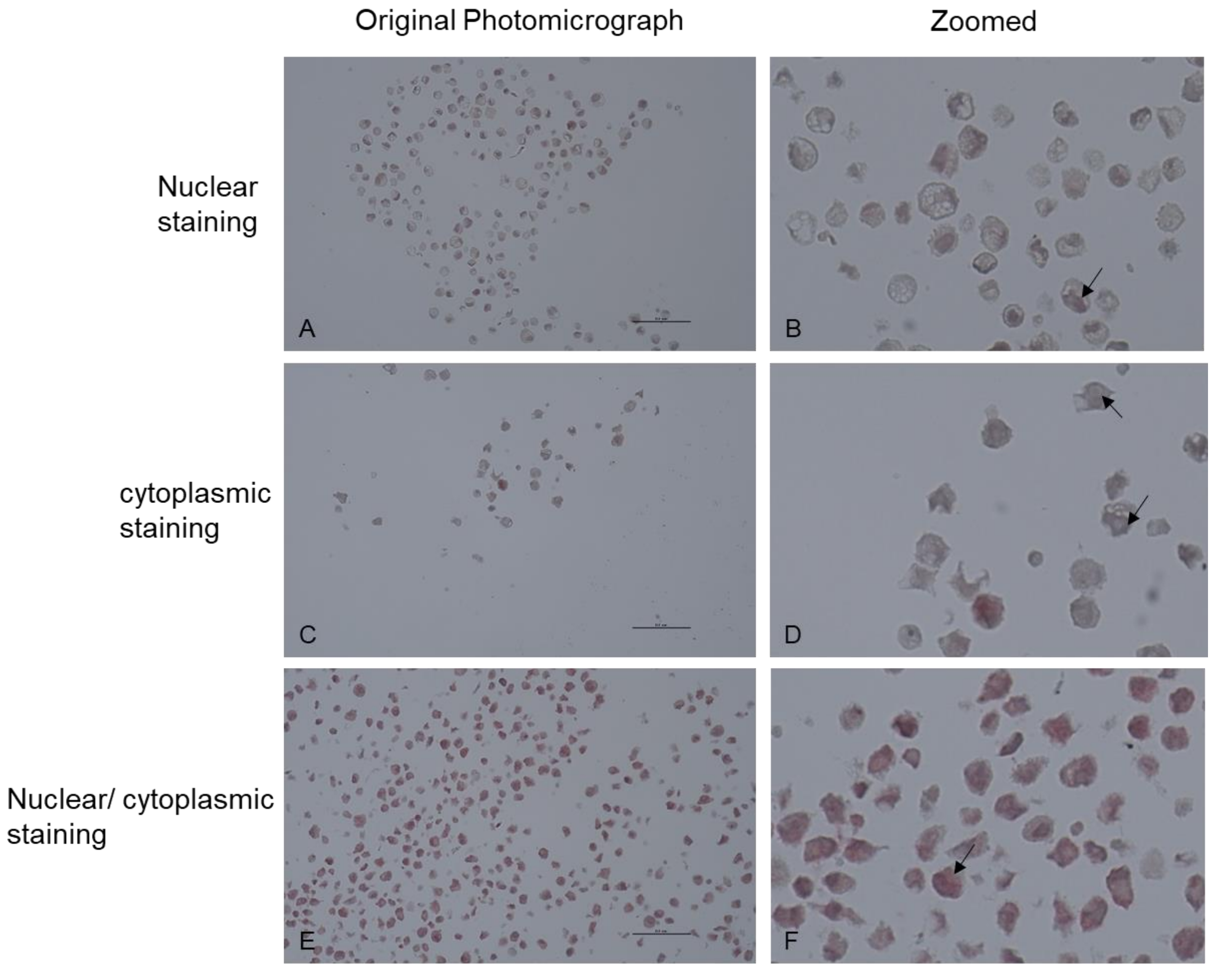

Supplementary Figure 1: Visual examples for cytoplasmic, nuclear or both staining of MT1/2 in cell lines. A/B: Cells representing nuclear staining of MT1/2. C/D: Cells representing cytoplasmic staining of MT1/2. E/F: Cells representing both nuclear and cytoplasmic staining of MT1/2. Stained cells are exemplarily indicated by arrows in zoomed photomicrographs (B, D and F).

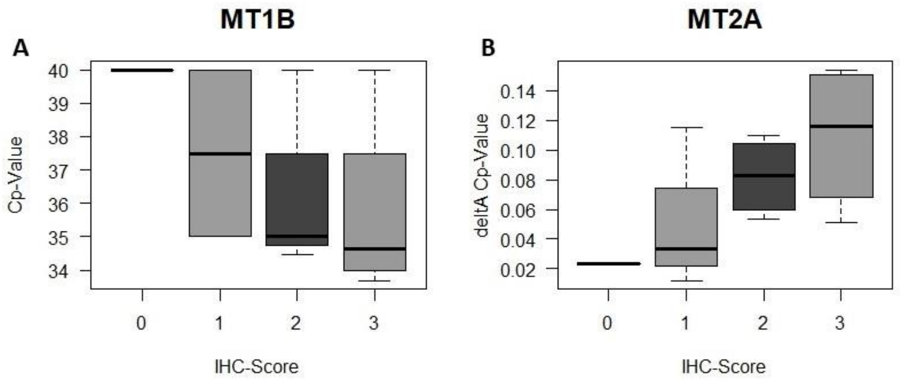

Suppl. Figure 2: Correlation between IHC-score and qPCR results of MT1B and MT2A in all three tested MPM cell lines. MT1B (A) showed proportional correlations, indicating that a strong mRNA expression of MT1B resulted in higher protein yields and thus correlated with a higher Score (p= 0.0012). This also applies to MT2A (B), as a higher ∆Cp-value correlated with higher scores (p<0.0001).

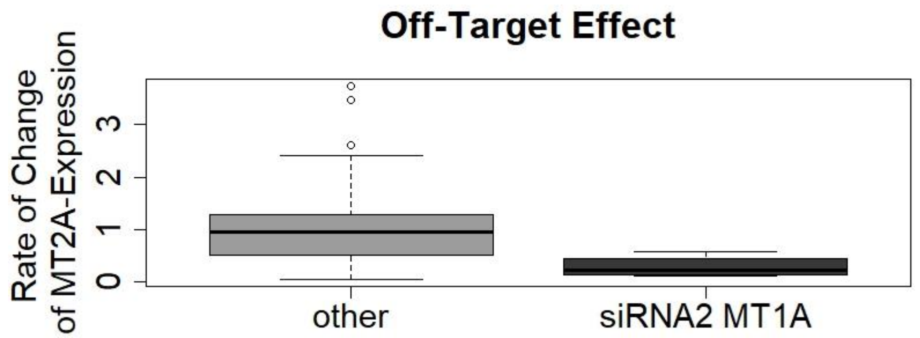

Suppl. Figure 3: Off-target effect of siRNA2 targeting MT1A. The siRNA2 targeting MT1A also targets MT2A significantly (p<0.0001). The p-value was calculated by using the Kruskal-Wallis test.
